# Core Endurance Relationships With Athletic and Functional Performance in Inactive People

**DOI:** 10.3389/fphys.2019.01490

**Published:** 2019-12-18

**Authors:** Marta Silva Santos, David G. Behm, David Barbado, Josimari Melo DeSantana, Marzo Edir Da Silva-Grigoletto

**Affiliations:** ^1^Physical Education Department, Functional Training Group, Graduate Program in Physiological Sciences, Federal University of Sergipe, São Cristóvão, Brazil; ^2^School of Human Kinetics and Recreation, Memorial University of Newfoundland, St. John’s, NF, Canada; ^3^Sport Research Centre, Department of Sport Sciences, Miguel Hernández University of Elche, Elche, Spain; ^4^Neuroscience Research Laboratory, Department of Physiotherapy, Graduate Program in Physiological Sciences, Federal University of Sergipe, São Cristóvão, Brazil

**Keywords:** chronic low back pain, athletic performance, rehabilitation, torso, muscles

## Abstract

Research regarding the relationship between core muscle endurance and performance is limited. The purpose of this study was to analyze the association between core/trunk endurance and athletic performance. Seventy-four healthy participants between 18 and 45 years old participated in this study (Age: 26.0 ± 6.5 years; Mass: 74.6 ± 12.8 kg; Height: 1.74 ± 0.08 m; BMI: 19.0 ± 6.8 kg/m^2^). The core endurance was measured using the McGill protocol, consisting of the following tests: trunk flexion, back extension, and side-bridge. Functional performance was evaluated with push-ups, sit to stand, T-run test, countermovement jump (CMJ), Yo-Yo test, maximum dynamic strength-one repetition maximum (1RM) and muscle power on the bench press, pull row, and leg press. The regression results between the McGill protocol (proxy for core/trunk endurance) and the dependent variables were: 1RM pull row: *r*^2^ = 0.109 with *p* = 0.046; RM bench press: *r*^2^ = 0.149 with *p* = 0.012; RM leg press: *r*^2^ = 0.144 with *p* = 0.013 and power pull row: *r*^2^ = 0.151 with *p* = 0.016; power bench press: *r*^2^ = 0.136 with *p* = 0.026; power leg press: *r*^2^ = 0.122 with *p* = 0.013), push-ups: *r*^2^ = 0.157 with *p* < 0.001, sit to stand: *r*^2^ = 0.198 with *p* < 0,001), functional movement score: *r*^2^ = 0.209 with *p* < 0.001). Nevertheless, core endurance scores were not able to predict jump ability (*r*^2^ = 0.014, *p* = 0.807) or agility (*T*-test: 0.036 with *p* = 0.497). In conclusion, core endurance exerted no significant influence the agility and jump performance but influenced the ability to run intermittently, exert maximum power and strength in different actions (push, pull, and lift exercises) related to the better quality of movement (FMS).

## Introduction

Core strength has been defined as the core or trunk muscles capacity to produce and maintain force (Vera-García et al., 2015). Based on this definition, core strength can be evaluated from the perspective of maximum strength (Shahtahmassebi et al., 2017), power (Shinkle et al., 2012) or even the ability to maintain force over time, which is called strength-endurance (McGill et al., 1999; Vera-García et al., 2015; Dello Iacono et al., 2016). Maintaining good endurance of the trunk and hip muscles (core) has been considered important for sports performance (Tong et al., 2014), injury prevention (Khaiyat and Norris, 2018), and rehabilitation (Willson et al., 2005; Dello Iacono et al., 2017). Theoretically, core endurance permits core stabilization for prolonged durations, which in turn, would facilitate acute and persistent force transmission and production during sports and daily life activities. This statement has been reinforced by experimental results, which have shown that core fatigue reduces the ability to produce shoulder strength during isometric contractions (Rosemeyer et al., 2015). These findings suggest that core fatigue hinders the stabilization of the core structures, which in turn, reduces the force production (Kibler et al., 2006; Silfies et al., 2015). However, it is still unclear to what extent core endurance could influence functional or athletic performance (Tse et al., 2005; Nesser et al., 2008; Tong et al., 2014). In this sense, the studies are contradictory when evaluating the relationship between core endurance and athletic or functional related parameters (Nesser et al., 2008; Tong et al., 2014). For example, in the elderly population, the morphology (cross-sectional area) (Sions et al., 2017), and muscle strength (Shahtahmassebi et al., 2017) of the core influenced functional ability of this population. However, Nesser et al. (2008) found positive but low to moderate correlations between core endurance and soccer players’ sports performance suggesting that core endurance training should not be an important target for the conditioning of this soccer athletes. It should also be noted that this relationship was not found in amateur athletes (Okada et al., 2011).

Based on this controversy, it seems plausible that core strength (maximal or endurance) can influence functional or athletic performance in different ways, depending on the population characteristics (athletes or sedentary, young or old individuals). Hence, core endurance could be a conditional capability, which only affects functional (Johnson et al., 2018) or deficit conditions (Cinar-Medeni et al., 2015). For this reason, data showe that the core endurance has a positive effect on the motor performance in physically inactive individuals (Okada et al., 2011; Tong et al., 2016). Based on this argument, a proper core fitness status could indeed be a determinant for functional outcomes in the sedentary population. However, most of the studies that seek to evaluate the influence of core endurance on performance use athletes as the sample population (Okada et al., 2011; Aytar et al., 2012; Tong et al., 2014, 2016). To our knowledge this hypothesis has not yet been tested with less active, more sedentary individuals. This research would help to understand the contribution of core endurance during activities of daily living, facilitating the prescription of training that seeks to improve/maintain the functionality of this population.

Despite this, it is hypothesized that core endurance plays a key role in providing greater stability to the average untrained individual (Franklin and Granata, 2007). Unlike athletes who seem to have adequate core endurance, the individuals with low physical activity levels probably have a lower core endurance level, which in turn may prevent the transfer of force from the center of the body to the extremities. Previous results with other populations such as those with chronic low back pain, where the core endurance deficit influences the functionality may confirm this idea (Vanti et al., 2016). Hence, there could be a relationship between core endurance and muscular performance, since endurance would provide an improvement in the core stability and therefore a more functional optimal kinetic chain optimization (distribution of force from the core to the extremities). Thus, to our knowledge, there are no studies that analyzed and compared the potential influence of core endurance on the muscular and athletic performance of underactive and young individuals. Therefore, the main objective of this study was to analyze the association between core endurance with functional and athletic performance in sedentary and inactive young individuals. It was hypothesized that core/trunk endurance would demonstrate significant and positive relationships with functional and athletic performance measures.

## Materials and Methods

### Participants

Endurance variables were considered for the power sample calculation. Based on *a priori* statistical power analysis (G-Power 3.1.9.4 for Windows, University of Düsseldorf) it was determined 64 participants would be needed to achieve an alpha of 0.05, power of 0.8 and an effect size of 0.5 (Khaiyat and Norris, 2018). Thus, 64 healthy young individuals between 18 and 45 years took part in this study: with 28 male participants (Age: 26.0 ± 6.5 years; Mass: 74.6 ± 12.8 kg; Height: 1.74 ± 0.08 m; BMI: 19.0 ± 6.8 kg/m^2^) and 36 female participants (Age: 23.8 ± 6.7 years; Mass: 64.6 ± 10.2 kg; Height: 1.62 ± 0.06 m; BMI: 25.0 ± 5.3 kg/m^2^). All participants were classified as inactive individuals according to the physical activity level questionnaire (Lee et al., 2011) as they did not perform any type of physical exercise in the last 3 months. Individuals who (a) suffered low back pain in the last 6 months, (b) ankle instability, (c) metabolic diseases (i.e., diabetes, hypertension, dyslipidemias), osteoarticular or musculoskeletal disorders were excluded from the study sample. This study was approved by the Regional Committee of the Federal University of Sergipe (CAAE: 68725017.3.0000.5546-053820/2017). The participants were voluntary and signed the informed consent form. All the research was in compliance with the Ethics Code of the World Medical Association (Helsinki Declaration).

### Measures

All the participants were informed about the expectations and procedures of the study as well as the test features. The tests were performed on two different days, 24 h apart. The tests were distributed over 2 days, so that the execution of one test would not interfere with the execution of another test. Thus, the tests that induced the most physical exhaustion were performed last. In addition, sufficient rest was given between each of the prior tests to ensure fatigue was not a factor. On the first day, participants performed the following tests in this sequence: countermovement jump (CMJ) followed by 20 min rest, T-run test (followed by 20 min rest), maximum dynamic strength (one repetition maximum, 1RM) of the bench press, pull row and leg press machine (followed by 40 min rest) and push-ups. On the second day, alternative tests were performed in the following order: muscular power (bench press, pull row, and leg press machine) and core endurance tests (front bridge, back extension, and side-bridge) with a 50 min rest between the sets of tests. Between the bench press, pull row and leg press tests, a 20 min recovery was provided. This occurred for both 1RM and power tests. The detailed description of each test can be found in a previous study except for the muscle power protocol (Santos et al., 2018).

### Design and Procedures

In this study, a multivariate regression design was applied to clarify the potential influence of core endurance on athletic and functional performance in young sedentary people. Specifically, tests of core isometric endurance (front, back, and side-bridge tests) were performed until task fatigue (inability to maintain correct posture). These tests were used as independent variables. The dependent variables were: functional movement screen (FMS), CMJ, sitting and standing up, push-ups in 1 min, *T*-test Agility, Yo-Yo maximum dynamic strength-1RM) and power in the bench press, leg press, and pull row machines.

Endurance of upper limb muscles was measured by counting the highest number of push-ups in a 1 min period. Hands were placed at shoulder width. Push-ups were performed with support at the feet or knees for men and women, respectively. An experienced evaluator controlled the elbow joint extension and flexion range of motion to 0–90°, respectively (Dhahbi et al., 2018; Zalleg et al., 2018). With the T-run test, individuals ran as fast as possible (9.14 m forward) and touched a cone (A), then sprinted right (cone B – 4.57 m) and then left (cone C – 4.57 m) (Padulo et al., 2016). After returning to cone A again, participants ran backward to the starting position. The participant completed a practice test followed by three attempts, with the shortest time considered for analysis. Times were recorded by two pairs of photocells (Timing System, Salt Lake City, UT, United States).

For muscle power evaluation, a load corresponding to the 50% of one maximal repetition (1RM) test was used with a bench press, pull row and leg press machines. A warm-up of 10 repetitions with 30% 1RM load was performed. Then, the participant was verbally encouraged to perform the concentric phase of the exercise at the highest possible speed. The angle of each test was 90° and it was controlled by an experienced physical education professional. A linear encoder (Ergotest Innovation AS^®^, Linear displacement sensor SKU 1260 MuscleLAb System, Norway) connected to an integrated data analysis system was used (Ergotest Innovation AS^®^, MuscleLAb System, Norway). The speed was used to calculate mean and maximum power using the Musclab^®^ as described previously (Padulo et al., 2015a, 2017; Migliaccio et al., 2018; Santos et al., 2018).

With the CMJ test, the participant performed squats to the hip and knee angles that they thought were comfortable. Then, the participant performed a rapid jump to maximum height on a contact platform (Probiotics Inc.^TM^, Huntsville, AL, United States) (Padulo et al., 2015b). There were three attempts with a rest period of 1 min between each attempt. The highest jump was considered for analysis.

The protocol of McGill et al. (1999) was used to evaluate the static resistance of the core, with three tests that evaluate trunk flexors, trunk extensors, and side bridge exercise. The individual was encouraged to remain in the position of each test for as long as possible, with time to task failure as the dependent variable. In the side bridge test, the mean between the right and left sides was used (Santos et al., 2018).

### Statistical Analysis

Descriptive statistics were performed for all data. The relationships between variables were determined using multiple regressions with a backward stepwise type to define the best statistical power predictor(s). Core endurance was represented by the interaction of the three tests (trunk flexion, trunk/back extension, and side-bridge) and used as an independent variable. Each athletic and functionally related variable was used as a dependent parameter in the model. Thus, it was analyzed whether the variable: endurance (modeled as the interaction of the tests: trunk flexion, trunk extension, and side-bridge) influenced each of the different athletic and functional parameters. In addition, a univariate regression was used to analyze separately how each of the trunk tests influenced the performance parameters. The program SPSS 20.0 (SPSS Inc., Chicago, IL, United States) was used for all the statistical analyses. The raw data supporting the conclusions of this manuscript will be made available by the authors, without reservation, to any interested researcher. The significant level was fixed at *p* < 0.05.

## Results

The multivariate regression identified that the union/sum of the three-predictor core endurance variables (McGill protocol) was able to exert a greater statistical power than the individual tests (i.e., trunk flexion, extension and side bridge tests) to explain the dependent variables (Table 1). Tables 2, 3 show the *r*^2^ values and *p*-value (significance) of the interaction between the core endurance and each functional performance variable. As shown in Tables 1–3, the relation to the other performance variables, there were statistically significant influences from 10.9 to 46.9%, with the greatest influence being the Yo-Yo test.

**TABLE 1:** Descriptive values of core variables and functional performances.

**Variables**	**Mean and SD**
Trunk flexion (s)	106.3 ± 41.8
Back extension (s)	94.0 ± 31.5
Side bridge (s)	50.4 ± 21.8
Functional movement screen (unitless)	13.2 ± 2.0
Countermovement jump (cm)	39.2 ± 19.6
Sit to stand (cm)	41.5 ± 7.4
Push-up (cm)	18.4 ± 9.7
Pull row (kg)	54.5 ± 20.7
Bench press (kg)	44.5 ± 21.1
Leg press (kg)	360.7 ± 100.5
Pull row (W)	223.3 ± 114.3
Bench press (W)	119.2 ± 84.2
Leg press (W)	756.5 ± 341.0
*T*-test agility (s)	13.40 ± 4.26
Yo-Yo test (m)	284.7 ± 147.8

**TABLE 2:** Association between core endurance model and dependent variables such as jump ability, functional movement screen, ability to lift, push-ups, agility, and cardiorespiratory capacity.

	**Variable**	***R*^2^**	**β**	***R*^2^ significance**	**Individual *R***
**CMJ (cm)**					
	Trunk flexion		0.006		0.096
Model	Back extension	0.014	0.015	0.807	0.003
	Side bridge		0.023		0.075
**FMS (score)**					
	Trunk flexion		0.007		0.043
Model	Back extension	0.209	0.001	0.001	0.096
	Side bridge		0.036		0.194
**Sit to stand**					
	Trunk flexion		0.013		0.003
Model	Back extension		0.033	<0.001	0.000
	Side bridge	0.198	0.171		0.171
**Push up (repetitions)**					
	Trunk flexion		0.049		0.075
Model	Back extension	0.157	0.034	<0.001	0.008
	Side bridge		0.149		0.126
***T*-test (s)**					
	Trunk flexion		0.002		0.011
Model	Back extension	0.036	0.017	0.497	0.028
	Side bridge		0.020		0.023
**Yo-Yo test (m)**					
	Trunk flexion		0.837		0.098
Model	Back extension	0.469	0.786	<0.001	0.017
	Side bridge		3.397		0.104

*CMJ, countermovement jump; FMS, functional movement screen.*

**TABLE 3:** Association between core endurance model and dependent variables: maximum dynamic strength and power.

	**Variable**	***R*^2^**	**B**	***R*^2^ significance**	**Individual *R***
**Pull row (kg)**					
	Trunk flexion		0.048		0.012
Model	Back extension	0.109	0.164	0.046	0.006
	Side bridge		0.294		0.065
**Bench press (kg)**					
	Trunk flexion		0.026		0.009
Model	Back extension	0.149	0.157	0.012	0.006
	Side bridge		0.334		0.096
**Leg press (kg)**					
	Trunk flexion		0.414		0.063
Model	Back extension	0.144	0.437	0.013	0.007
	Side bridge		1.472		0.121
**Pull row (W)**					
	Trunk flexion		0.396		0.036
Model	Back extension	0.152	0.763	0.016	0.024
	Side bridge		1.914		0.121
**Bench press (W)**					
	Trunk flexion		0.257		0.018
Model	Back extension	0.136	0.707	0.026	0.003
	Side bridge		1.299		0.087
**Leg press (W)**					
	Trunk flexion		0.414		0.011
Model	Back extension	0.122	0.437	0.013	0.000
	Side bridge		1.432		0.099

The CMJ, *T*-test agility and pull-row were not significantly influenced by the core endurance tests, but trunk endurance influenced the variables: FMS (20.9%, *p* = 0.001), Sit to Stand (19.8%, *p* < 0.001), Push up (15.7%, *p* < 0,001), and Yo-Yo (0.46%, *p* < 0.001) (Table 2).

Regarding the strength measures (Table 3), there was a significant influence on the variables: pull row (10.9%, *p* = 0.046), bench press (14.9%, *p* = 0.012), and leg press (14.4, *p* = 0.013). Regarding the power measures there was significant influence on the variables: pull row (15.2%, *p* = 0.016), bench press (13.16%, *p* = 0.026), and leg press (12.2, *p* = 0.013).

## Discussion

Previous studies have suggested that greater core strength can facilitate force transmission from the proximal body segments (i.e., trunk or core) to the extremities, increasing motor efficiency (Hibbs et al., 2008; Silfies et al., 2015; Tong et al., 2016; Vega Toro et al., 2016). However, this relationship has not been well established by previous research. Although we did not directly evaluate the possible contributions of the core to segmental movement, this study provides an analysis of the potential that core endurance influences more complex neuromotor activities, such as running, jumping, pushing, among others. The main findings of this study were that the endurance of the core is related to the individuals’ ability to run intermittently and in general to have a better quality of movement (i.e., FMS). Despite this, core endurance was not able to predict jump ability and agility.

The main finding of this work showed that, unlike previous works (Nesser et al., 2008; Okada et al., 2011), core endurance displayed a significant, positive association with some athletic and functional tests. These results can be explained by two reasons. As far as we know, this study was the first to evaluate the potential influence of core endurance on athletic and functional-related measures avoiding the potential bias induced by bivariate correlational analyses. Several studies investigating the relationship between core endurance and performance also used the McGill protocol (Nesser et al., 2008; Okada et al., 2011; Ambegaonkar et al., 2016). This test quantifies the ability of the muscles to sustain a prolonged contraction in different planes being sensitive to detect endurance improvement caused by core training programs (Durall et al., 2009; Teyhen et al., 2013; Allen et al., 2014). Nevertheless, unlike most studies that performed bivariate Pearson’s correlations, we have carried out a multiple regression analysis, which permits a proper analysis of the global core endurance contribution for each athletic and functionally related measure. This global core endurance is assessed by the interaction between trunk flexion, extension, and side-bridge tests, which represent the anterior, posterior and lateral trunk muscles, respectively. On the other hand, the significant association found between core endurance and motor performance could be explained by the characteristics of our sample in which participants were classified as inactive young individuals.

The role of core endurance as a determinant parameter for functional independence was confirmed by the significant association between core parameters and functional performance tests. This is the case with the tests that evaluated the functionality, either in the perspective of how the individual moves (20.9%), how they sit and stand from a chair (sit to stand, 19.8%) or push-up from the ground (15.7%). Regarding the quality of movement, Okada et al. (2011) stated that there was no correlation between the seven movements of the FMS and the trunk flexion, extension, and side-bridge tests. However, while the total FMS score was not used, the study sample from Okada et al. (2011) was recreational athletes, which may partially explain the difference between the results. This can be ratified when analyzing the interaction between the three tests that compose the McGill tests in this study. There is greater predictive power for the quality of movement, when the three tests are evaluated as a unit score. When analyzing the core muscles separately (Table 2), each muscle influenced the quality of movement differently, with the lateral trunk flexors the most influential. However, the positive results from the multiple regression model suggest that when the individual moves (quality of movement), there is a synergic interaction between the core muscles and movement of the upper and lower limbs.

The muscular strength of lower limbs was evaluated with one of the most functional human actions; to sit and stand. Roldan-Jimenez et al. (2015) evaluated three different conditions of the sit-to-stand test. In the first and second condition, 5–10 repetitions were performed, respectively, both occurring at a rate of 40 beats per minute (controlled by a metronome). The last condition was the maximum number of repetitions in 30 s. They observed that in the last condition, there was an increase in the muscular activation of the erector spine, and in all conditions, the tibialis anterior (23–26%), quadriceps (20–21%), rectus abdominis (17–18%), and erector spinae (10%) were the muscles that participated most in this sitting and standing from the chair. This finding shows that with the overload of only the body mass, there is a contribution to the global core muscles from 30 s of sitting and standing. In this sample, the sit-to-stand test lasted for one minute, which probably emphasized muscular endurance.

Regarding the significant association found between the McGill’s test and push-up test, pull-row, and bench press, our results seem to confirm previous findings, which suggested the relevance of core endurance as a pertinent factor to maintain proper upper-body performance over time. These results support previous experimental findings, which showed that core fatigue reduces the ability to produce upper-body strength (Rosemeyer et al., 2015). Interestingly, the push-up test showed a slightly higher association with core endurance than the bench press test. These results could be related to previous findings showing that core muscle activation is higher during push-up than bench press test. Thus, Calatayud et al. (2014) identified that the rectus abdominis muscle was activated at approximately 20% of MVIC (maximal voluntary isometric maximum contraction) in the young people during the push-up test. This activation was greater when compared to the bench press exercise (85% of 1RM Calatayud et al., 2014), demonstrating the functional role of the rectus abdominis in securing the viscera when the trunk is submitted to a greater intra-abdominal pressure. With push-ups, the trunk or core is not supported to the same extent as with the bench press.

The results of the association between the core endurance showed the highest significant results with the Yo-Yo IR (46.9%) but not for *T*-test (3.6%). These findings differ from other recent articles (Kubo et al., 2011; Shinkle et al., 2012), which have investigated the relationship between the core endurance and the action of running. Tong et al. (2016) and collaborators evaluated the core endurance of amateur runners with a static test (specific endurance plank test performance). Their results showed that core endurance influenced 47.1% of the running economy and 32.5% of the performance of a 1-h running performance test. Kubo et al. (2011) associated the cross-sectional area of the trunk muscles with a 20-m sprint and observed that the lumbar-sacral and erector spine muscles had a significant influence on this type of race. These findings assume that the time and magnitude the core muscles usage during the run is influenced by the time or distance in which the individual needs to remain during this action, and thus with distances less than 20 m, as in the case of the *T*-test, core endurance is not a major requirement. However, this result should be viewed with caution, since it can be influenced by other factors such as the running technique. Another variable that evaluated lower limbs power was the CMJ, in which the stretch-shortening cycle was used to help generate the maximum power during the execution of a jump. Since the influence of endurance is negligible with a CMJ, there was no significant correlation, which is in accord with Nesser et al. (2008).

As a limitation, it must be noted that core fitness variables (endurance, strength, stability) can differently influence motor performance according to the characteristics of each population. According to Shinkle et al. (2012) and the concept of training and testing specificity (Behm and Sale, 1993), given the dynamic and intermittent characteristic of athletic performance, it would be expected the static core endurance tests may not be sensitive enough to measure the role of core function. However, in this study, unlike the population of healthy athletes, even when endurance was evaluated with static tests, it was able to predict the functional and athletic performance of young but sedentary adults. This seems to confirm that the core relevance is manifested in different ways according to the population characteristics (i.e., active versus inactive adults). However, this test might not be useful in other sport populations and in tests that dynamically evaluate the trunk.

## Conclusion

In conclusion, core endurance exerted no significant influence on the agility and ability to jump, but it influenced the ability of the subject to run intermittently (46.9%), exert maximum force (between 10.9 and 14.9%) and power (between 13.6 and –15.1%), push-ups (15.7%), sitting and lifting (19.8%). In addition, individuals with higher core endurance had a better quality of movement (20.9%).

## Data Availability Statement

The raw data supporting the conclusions of this article will be made available by the authors, without undue reservation, to any qualified researcher.

## Ethics Statement

This study was approved by the Regional Committee of the Federal University of Sergipe (CAAE: 68725017.3.0000.5546- 053820/2017). The participants were voluntary and signed the informed consent form. All the research was in compliance with the Ethics Code of the World Medical Association (Helsinki Declaration).

## Author Contributions

MS conceptualized, designed, analyzed, and interpreted the data, and drafted the manuscript and revised it critically for important intellectual content. DBa critically revised the manuscript for important intellectual content. JD and MD critically revised the manuscript. All authors approved the final manuscript and agreed to be accountable for all aspects of the work.

## Conflict of Interest

The authors declare that the research was conducted in the absence of any commercial or financial relationships that could be construed as a potential conflict of interest.

## References

[B1] Allen B. A. Hannon J. C. Burns R. D. Williams S. M. (2014). Effect of a core conditioning intervention on tests of trunk muscular endurance in school-aged children. *J. Strength Cond. Res.* 28 2063–2070. 10.1519/jsc.0000000000000352 24378666

[B2] Ambegaonkar J. P. Cortes N. Caswell S. V. Ambegaonkar G. P. Wyon M. (2016). Lower extremity hypermobility, but not core muscle endurance influences balance in female collegiate dancers. *Int. J. Sports Phys. Ther.* 11 220–229. 10.12678/1089-313x.22.2.75 27104055 PMC4827365

[B3] Aytar A. Pekyavas N. O. Ergun N. Karatas M. (2012). Is there a relationship between core stability, balance and strength in amputee soccer players? a pilot study. *Prosthet. Orthot. Int.* 36 332–338. 10.1177/0309364612445836 22918911

[B4] Behm D. G. Sale D. G. (1993). Velocity specificity of resistance training: a review. *Sports Med.* 15 374–388. 10.2165/00007256-199315060-00003 8341872

[B5] Calatayud J. Borreani S. Colado J. C. Martin F. Rogers M. E. (2014). Muscle activity levels in upper-body push exercises with different loads and stability conditions. *Phys. Sportsmed.* 42 106–119. 10.3810/psm.2014.11.2097 25419894

[B6] Cinar-Medeni O. Baltaci G. Bayramlar K. Yanmis I. (2015). Core stability, knee muscle strength, and anterior translation are correlated with postural stability in anterior cruciate ligament-reconstructed patients. *Am. J. Phys. Med. Rehabil.* 94 280–287. 10.1097/phm.0000000000000177 25122103

[B7] Dello Iacono A. Maffulli N. Laver L. Padulo J. (2017). Successful treatment of groin pain syndrome in a pole-vault athlete with core stability exercise. *J. Sports Med. Phys. Fitness* 57 1650–1659. 10.23736/s0022-4707.16.06735-9 27792221

[B8] Dello Iacono A. Padulo J. Ayalon M. (2016). Core stability training on lower limb balance strength. *J. Sports Sci.* 34 671–678. 10.1080/02640414.2015.1068437 26177151

[B9] Dhahbi W. Chaabene H. Chaouachi A. Padulo J. Behm D. Cochrane J. (2018). Kinetic analysis of push-up exercises: a systematic review with practical recommendations. *Sports Biomech.* 4 1–40. 10.1080/14763141.2018.1512149 30284496

[B10] Durall C. J. Udermann B. E. Johansen D. R. Gibson B. Reineke D. M. Reuteman P. (2009). The effects of preseason trunk muscle training on low-back pain occurrence in women collegiate gymnasts. *J. Strength Cond. Res.* 23 86–92. 10.1519/JSC.0b013e31818b93ac 19057402

[B11] Franklin T. C. Granata K. P. (2007). Role of reflex gain and reflex delay in spinal stability–a dynamic simulation. *J. Biomech.* 40 1762–1767. 10.1016/j.jbiomech.2006.08.007 17054964

[B12] Hibbs A. E. Thompson K. G. French D. Wrigley A. Spears I. (2008). Optimizing performance by improving core stability and core strength. *Sports Med.* 38 995–1008. 10.2165/00007256-200838120-00004 19026017

[B13] Johnson C. D. Whitehead P. N. Pletcher E. R. Faherty M. S. Lovalekar M. T. Eagle S. R. (2018). The relationship of core strength and activation and performance on three functional movement screens. *J. Strength Cond. Res.* 32 1166–1173. 10.1519/jsc.0000000000001943 28445228

[B14] Khaiyat O. A. Norris J. (2018). Electromyographic activity of selected trunk, core, and thigh muscles in commonly used exercises for ACL rehabilitation. *J. Phys. Ther. Sci.* 30 642–648. 10.1589/jpts.30.642 29706723 PMC5909019

[B15] Kibler W. B. Press J. Sciascia A. (2006). The role of core stability in athletic function. *Sports Med.* 36 189–198. 10.2165/00007256-200636030-00001 16526831

[B16] Kubo T. Hoshikawa Y. Muramatsu M. Iida T. Komori S. Shibukawa K. (2011). Contribution of trunk muscularity on sprint run. *Int. J. Sports Med.* 32 223–228. 10.1055/s-0030-1268502 21165810

[B17] Lee P. H. Macfarlane D. J. Lam T. H. Stewart S. M. (2011). Validity of the international physical activity questionnaire short form (IPAQ-SF): a systematic review. *Int. J. Behav. Nutr. Phys. Act.* 8:115. 10.1186/1479-5868-8-115 22018588 PMC3214824

[B18] McGill S. M. Childs A. Liebenson C. (1999). Endurance times for low back stabilization exercises: clinical targets for testing and training from a normal database. *Arch. Phys. Med. Rehabil.* 80 941–944. 10.1016/s0003-9993(99)90087-4 10453772

[B19] Migliaccio G. M. Dello Iacono A. Ardigo L. P. Samozino P. Iuliano E. Grgantov Z. (2018). Leg press vs. smith machine: quadriceps activation and overall perceived effort profiles. *Front. Physiol.* 9:1481. 10.3389/fphys.2018.01481 30405437 PMC6206431

[B20] Nesser T. W. Huxel K. C. Tincher J. L. Okada T. (2008). The relationship between core stability and performance in division i football players. *J. Strength Cond. Res.* 22 1750–1754. 10.1519/JSC.0b013e3181874564 18978631

[B21] Okada T. Huxel K. C. Nesser T. W. (2011). Relationship between core stability, functional movement, and performance. *J. Strength Cond. Res.* 25 252–261. 10.1519/JSC.0b013e3181b22b3e 20179652

[B22] Padulo J. Bragazzi N. L. Nikolaidis P. T. Dello Iacono A. Attene G. Pizzolato F. (2016). Repeated sprint ability in young basketball players: multi-direction vs. one-change of direction (Part 1). *Front. Physiol.* 7:133. 10.3389/fphys.2016.00133 27148072 PMC4840326

[B23] Padulo J. Laffaye G. Chaouachi A. Chamari K. (2015a). Bench press exercise: the key points. *J. Sports Med. Phys. Fitness* 55 604–608. 24823345

[B24] Padulo J. Tabben M. Attene G. Ardigo L. P. Dhahbi W. Chamari K. (2015b). The impact of jumping during recovery on repeated sprint ability in young soccer players. *Res. Sports Med.* 23 240–252. 10.1080/15438627.2015.1040919 26038845

[B25] Padulo J. Migliaccio G. M. Ardigo L. P. Leban B. Cosso M. Samozino P. (2017). Lower limb force, velocity, power capabilities during leg press and squat movements. *Int. J. Sports Med.* 38 1083–1089. 10.1055/s-0043-118341 29050041

[B26] Roldan-Jimenez C. Bennett P. Cuesta-Vargas A. I. (2015). Muscular activity and fatigue in lower-limb and trunk muscles during different sit-to-stand tests. *PLoS One* 10:e0141675. 10.1371/journal.pone.0141675 26506612 PMC4624782

[B27] Rosemeyer J. R. Hayes B. T. Switzler C. L. Hicks-Little C. A. (2015). Effects of core-musculature fatigue on maximal shoulder strength. *J. Sport Rehabil.* 24 384–390. 10.1123/jsr.2014-0216 25658299

[B28] Santos M. S. Vera-Garcia F. J. Da Silva Chavesa L. M. Brandão L. A. Da Silva D. R. P. Da Silva-Grigoletto M. E. (2018). Are core exercises important to functional training protocols? *Rev. Andal. Med. Deporte* 11 237–244. 10.33155/10.33155/j.ramd.2018.02.002

[B29] Shahtahmassebi B. Hebert J. J. Hecimovich M. D. Fairchild T. J. (2017). Associations between trunk muscle morphology, strength and function in older adults. *Sci. Rep.* 7:10907. 10.1038/s41598-017-11116-0 28883555 PMC5589953

[B30] Shinkle J. Nesser T. W. Demchak T. J. McMannus D. M. (2012). Effect of core strength on the measure of power in the extremities. *J. Strength Cond. Res.* 26 373–380. 10.1519/JSC.0b013e31822600e5 22228111

[B31] Silfies S. P. Ebaugh D. Pontillo M. Butowicz C. M. (2015). Critical review of the impact of core stability on upper extremity athletic injury and performance. *Braz. J. Phys. Ther.* 19 360–368. 10.1590/bjpt-rbf.2014.0108 26537806 PMC4647147

[B32] Sions J. M. Elliott J. M. Pohlig R. T. Hicks G. E. (2017). Trunk muscle characteristics of the multifidi, erector spinae, psoas, and quadratus lumborum in older adults with and without chronic low back pain. *J. Orthop. Sports Phys. Ther.* 47 173–179. 10.2519/jospt.2017.7002 28158957 PMC7064314

[B33] Teyhen D. S. Childs J. D. Dugan J. L. Wright A. C. Sorge J. A. Mello J. L. (2013). Effect of two different exercise regimens on trunk muscle morphometry and endurance in soldiers in training. *Phys. Ther.* 93 1211–1224. 10.2522/ptj.20120152 23064733

[B34] Tong T. K. McConnell A. K. Lin H. Nie J. Zhang H. Wang J. (2016). Functional inspiratory and core muscle training enhances running performance and economy. *J. Strength Cond. Res.* 30 2942–2951. 10.1519/jsc.0000000000000656 25162653

[B35] Tong T. K. Wu S. Nie J. Baker J. S. Lin H. (2014). The occurrence of core muscle fatigue during high-intensity running exercise and its limitation to performance: the role of respiratory work. *J. Sports Sci. Med.* 13 244–251. 24790475 PMC3990875

[B36] Tse M. A. McManus A. M. Masters R. S. (2005). Development and validation of a core endurance intervention program: implications for performance in college-age rowers. *J. Strength Cond. Res.* 19 547–552. 10.1519/15424.1 16095402

[B37] Vanti C. Conti C. Faresin F. Ferrari S. Piccarreta R. (2016). The relationship between clinical instability and endurance tests, pain, and disability in nonspecific low back pain. *J. Manipulative Physiol. Ther.* 39 359–368. 10.1016/j.jmpt.2016.04.003 27167368

[B38] Vega Toro A. S. Cools A. M. de Oliveira A. S. (2016). Instruction and feedback for conscious contraction of the abdominal muscles increases the scapular muscles activation during shoulder exercises. *Man. Ther.* 25 11–18. 10.1016/j.math.2016.05.331 27422592

[B39] Vera-García F. J. Barbado D. Moreno-Pérez V. Hernández-Sánchez S. Juan-Recio C. Elvira J. L. L. (2015). Core stability. concepto y aportaciones al entrenamiento y la prevención de lesiones. *Rev. Andal. Med. Deporte* 8 79–85. 10.1016/j.ramd.2014.02.004

[B40] Willson J. D. Dougherty C. P. Ireland M. L. Davis I. M. (2005). Core stability and its relationship to lower extremity function and injury. *J. Am. Acad. Orthop. Surg.* 13 316–325. 10.5435/00124635-200509000-00005 16148357

[B41] Zalleg D. Ben Dhahbi A. Dhahbi W. Sellami M. Padulo J. Souaifi M. (2018). Explosive push-ups: from popular simple exercises to valid tests for upper-body power. *J. Strength Cond. Res.* 10.1519/jsc.0000000000002774 [Epub ahead of print]. 30095736

